# Reconstruction of American Women in State and Territorial Legislative Offices, 1895–1995

**DOI:** 10.1016/j.dib.2024.110631

**Published:** 2024-06-18

**Authors:** Gregori Galofré-Vilà

**Affiliations:** Department of Economic Analysis, Universitat de València, Spain

**Keywords:** Female legislators, Political economy, Voting rights, U.S. government, United States

## Abstract

This data paper presents a reconstruction of American women who have served in state and territorial legislatures (female legislators). The dataset spans from 1895 to 1995 and is reported at an annual basis. For all 6,466 women, individual information on each female legislator is provided, including their name, surname, party affiliation, city and county of residence, and the state they represented. Data for the Senate and House are reported separately. The data was extracted from the encyclopedia titled ``Women State and Territorial Legislators, 1895–1995. A State-by-State Analysis, with Rosters of 6,000 Women'' [1].

The dataset can be used to study patterns in political representation, assessing the involvement of women in government, and delving into significant themes such as the intersection between women legislators and the historical, cultural, and political dynamics of their era. The categorization of women according to their city/county of residence enables researchers to seamlessly integrate this data with other spatio-temporal databases. Additionally, the dataset includes the FIPS county codes corresponding to each woman's residence, facilitating convenient linkage with other datasets, such as census data, using the FIPS code.

Specifications Table

**Table:** 

Subject	Political Science
Specific subject area	Data from "Women State and Territorial Legislators" details 6466 American women in state and territorial legislatures (1895–1995), aiding political representation analysis.
Type of data	Raw data, Panel data.
Data collection	Data were manually collected and digitized and inputted into an Excel spreadsheet.
Data source location	Data were collected from [1]
Data accessibility	Repository name: Mendeley Data identification number: DOI: 10.17632/w7gwsfbz89.1 Direct URL to data: https://data.mendeley.com/datasets/w7gwsfbz89/1 Instructions for accessing these data: Data available on Mendeley. Published online 20 March 2024.

## 1 Value of the Data

• The data aims to enhance comprehension of women's involvement in politics spanning from 1895 to 1995.
• The data provides contextual information for understanding political and cultural U.S. history.
• The data constitutes an annual, comprehensive panel of women in U.S. politics, offering detailed information on their political affiliations and residence.
• The categorization of women by county FIPS number enables flexible linkage with other spatial datasets.
• The dataset is pertinent for all social scientists, including economists, political scientists, and historians, who are interested in studying political behavior in the U.S.

## 2 Background

The creation of the dataset stems from the necessity to fill a gap in historical and political research regarding the participation of American women in state and territorial legislative offices. The dataset aims to provide comprehensive information on female legislators over a century. Theoretical and methodological considerations underpinning the compilation of this dataset likely include a feminist perspective seeking to address the historical underrepresentation of women in politics. This dataset facilitates empirical analyses of patterns in political representation, allowing researchers to explore the evolving role of women in government. Moreover, by including detailed individual-level data such as party affiliation and geographic information, the dataset enables nuanced examinations of the intersection between gender and various historical, cultural, and political dynamics. It also enables to explore if having a higher share of female legislators allowed for more social policies in terms of health and education.

## 3 Data Description

The data comes from the encyclopedia titled the “Women State and Territorial Legislators" [1]. The raw data extracted from [1] was manually processed and cleaned. The resulting output file is available in Stata 16. The file contains information on each female state legislator, including their city and county of residence, party affiliation, and the years they were elected. Notes for each variable are included within the dataset.

**Fig. 1:**
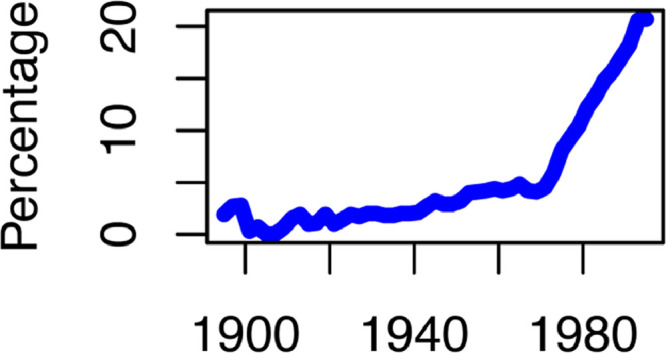

Fig. 1 shows the share of state women legislators over time. The data spans from the first 3 women serving in Colorado in 1895 to 1535 women in 1995. Notably, prior to 1920, female representation remained low, constituting less than 2 % of all legislators. However, between 1920 and 1969, women started to gain popularity, but still representing approximately less than 5 % of state legislators. Their share started to increase with the enactment of the Voting Rights Act of 1965, which granted voting and political rights to disenfranchised African Americans in the South, impacting on the gender composition of state legislatures, and more profoundly with the passage of the Equal Rights Amendment by the U.S. Congress in 1972, which facilitated greater female participation in state legislative politics. While unreported here, there is also a wide range of spatial heterogeneity, with states like Connecticut, Vermont, and New Hampshire boasting over 400 female state legislators, while Alabama, Louisiana, Delaware, Nebraska, and Arkansas had fewer than 50.

## 4 Experimental Design, Materials and Methods

The raw data extracted from [1] was manually processed and cleaned. The resulting output file is available in Stata 16. The file contains information on each female state legislator, including their city and county of residence, party affiliation, and the years they were elected. Notes for each variable are included within the dataset Table 1.

**Table 1:** Content of the related field dataset.

Variable	Variable Content
Place	It denotes if it served in the house or senate. Forty-nine states feature bicameral legislatures, typically consisting of a lower chamber known as the House and an upper chamber, the Senate. Exceptions include New York, New Jersey, or Nebraska.
Years	Most states hold legislative elections in even-numbered years, with legislative sessions commencing in the subsequent odd years. This two-year cycle, from one November to the next in even years, is commonly referred to as the election cycle. Conversely, the two years from January to January in odd years constitute the legislative cycle. Nonetheless, six states deviate from this pattern, including Mississippi, New Jersey, and Virginia, where legislative elections occur in odd years and sessions convene in even years.
Name	This field comprises the first name and surname of each woman legislator. Women are listed with their given first name and their surname at the time of their service. This distinction is significant as married women were often identified by their husband's names.
Fips	Federal Information Processing System (FIPS) Codes for U.S. Counties. FIPS codes are numbers which uniquely identify geographic areas. For details see
State	State's name
County	County's name.
City	City's name
Party	Party Affiliation. All women legislators are recorded with their party affiliation, mostly Republican and Democratic, and also including Nonpartisan and Independent designations and women who served under minor party labels such as Populist, Progressive, Farm Labor or Socialist.
i_1 – i_26	Years in office.

## Limitations

None.

## Ethics Statement

The work did not involve the use of human subjects, animal experiments or data collected from social media platforms.

## Credit Author Statement

This is a single-authored article: Conceptualization, Investigation, Data curation, Writing–review & editing.

## Data Availability

Female legislators (Original data) (Mendeley Data).

## References

[1] Cox Elizabeth M. (1996).

